# Infiltrative Intramuscular Lipoma with Overlying Fascial Defect in the Anterior Thigh: A Case Report

**DOI:** 10.7759/cureus.56274

**Published:** 2024-03-16

**Authors:** Tristen N Taylor, Richard D Murray, Dillon L Morrow, Joseph Duff, Ryan T Voskuil

**Affiliations:** 1 Department of Orthopaedic Surgery, Baylor College of Medicine, Houston, USA; 2 Department of Orthopaedic Surgery, University of Tennessee Health Science Center College of Medicine, Chattanooga, USA

**Keywords:** intramuscular lipoma, defect, fascia, infiltrative lipoma, lipoma

## Abstract

This case report details a unique presentation of an infiltrative intramuscular lipoma in the anterior thigh of a 51-year-old female with an overlying fascial defect. The patient reported a progressively enlarging left thigh mass associated with pain exacerbated by knee movement and exercise. MRI revealed a homogeneous intramuscular lipoma without contrast enhancement with a fascial defect. An 8 cm longitudinal incision exposed a 7 x 4 cm fascial defect overlying the lipomatous mass within the rectus femoris muscle. Pathological analysis confirmed an intramuscular lipoma without malignancy. Follow-ups at two, six, and 12 weeks demonstrated pain resolution and no soft tissue bulge. This case underscores the importance of distinguishing intramuscular lipomas from other neoplasms, such as lipomatosis and liposarcomas. The association of a fascial defect with intramuscular lipomas is unprecedented and may be due to the increased pressure on the fascia by the lipoma. The report emphasizes the role of MRI in diagnosis and appropriate surgical management, and highlights the need for further exploration into the etiology of fascial defects associated with intramuscular lipomas.

## Introduction

Lipomas are typically benign, subcutaneous, mesenchymal tumors composed of mature lipocytes, most commonly located superficial to the fascia [1]. Lipomas located deep to the fascia are termed subfascial lipomas, and deep-seated lipomas that are located between the musculature are referred to as intermuscular lipomas [2,3]. Lipomas presenting within musculature are termed intramuscular lipomas and account for less than 1% of all lipomas [2,3].

Histologically, intramuscular lipomas may present as infiltrative, well-defined/non-infiltrative, and mixed infiltration [2,4]. Infiltrative intramuscular lipomas are characterized by mature adipocytes with irregular interlacing of muscular fibers [3]. Our literature review found no reports of intramuscular lipomas with overlying fascial defect or deterioration. Therefore, we report on a case of a patient with a large, infiltrative, intramuscular lipoma with an associated fascial defect, which we hypothesize is due to the added pressure of the underlying lipoma.

## Case presentation

The patient was a 51-year-old female with a body mass index of 24.5 kg/m^2 ^who presented with a left thigh mass that she first noticed several years prior to presentation that had recently increased in size. She endorsed mild pain exacerbated by flexion and extension of the knee, weight-bearing, and exercise. She denied any history of direct trauma to the area, regional cutaneous anesthesia, or differences in perfusion or pulses between the two legs. Physical exam was significant for a palpable deep-seated mass that became more prominent during knee extension and quadriceps contraction. 

**Figure 1:**
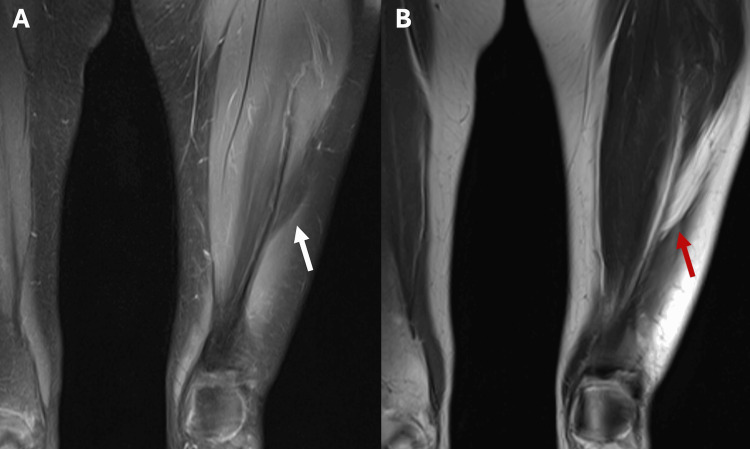
MRI images of the intramuscular lipoma within the patient's left leg (A) T2 weighted MRI showing non-enhancing mass (white arrow) within the vastus lateralis; (B) T1 fat-saturated MRI image showing homogenous isoenhancing mass (red arrow) to surrounding fat.

MRI revealed a 5.7 x 0.8 x 10.8 cm plaque-like, homogeneous mass located superficially in the anterior muscle compartment of the left thigh, approximately 10 cm superior to the superior pole of the patella, deep to fascia without contrast enhancement, as well as mild vastus lateralis irregularity (Figure 1). 

**Figure 2:**
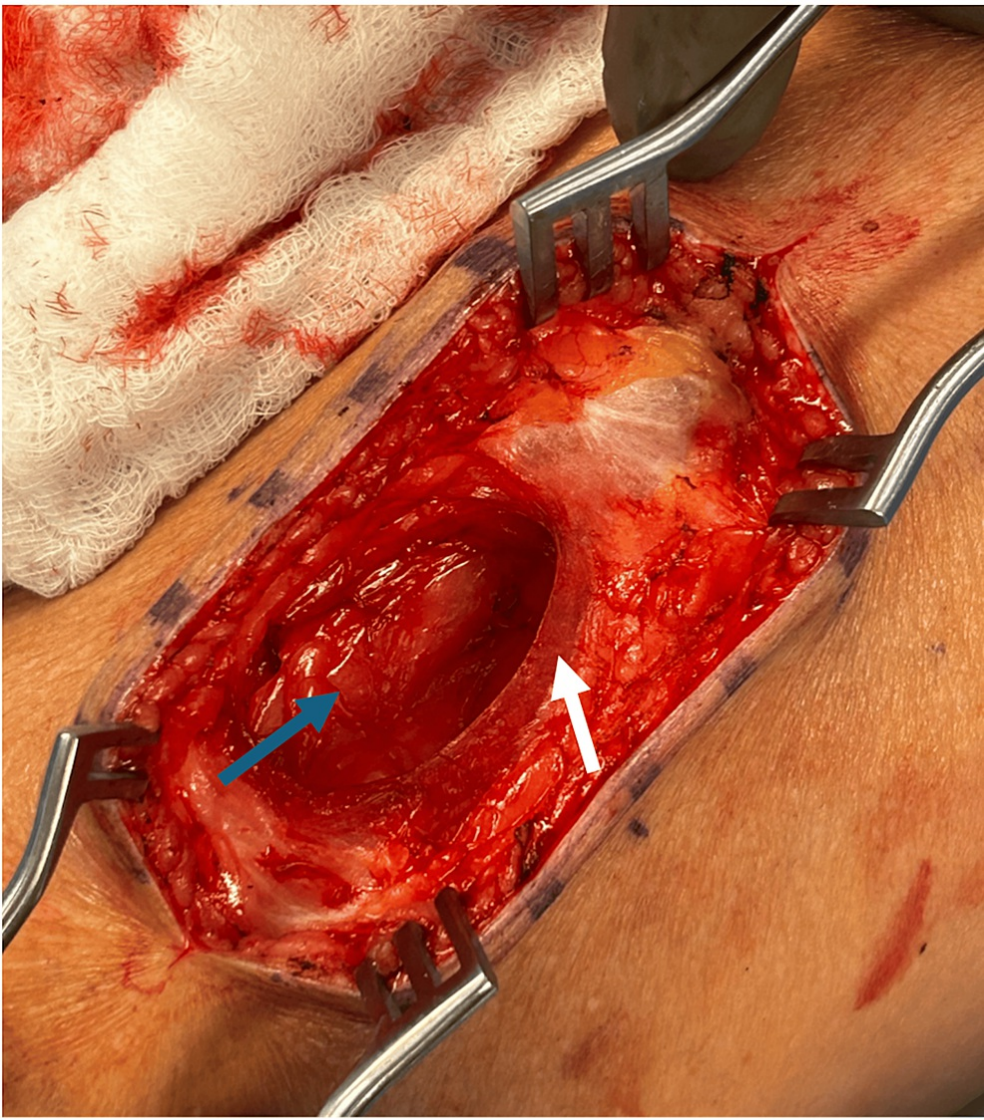
Fascial defect overlying the intramuscular lipoma Superficial to the intramuscular lipoma (blue arrow) is the fascial defect, indicated by the rounded edge of the fascia of the mid-anterior thigh (white arrow).

Due to progressive pain and limitation in activities of daily living despite conservative measures, the patient elected to proceed with surgical intervention. The left lower extremity was sterilely prepped and draped in standard fashion. An 8 cm longitudinal incision was made directly over the mass with a 10-blade scalpel. After dissecting to the fascial layer, a defect in the fascia was identified, measuring 7 x 4 cm, with palpable medial and lateral edges (Figure 2). The fascial defect was extended longitudinally, allowing full visualization of the fatty tumor within the rectus femoris muscle belly. The fatty mass had a plaque-like appearance and less of a discrete mass within its own capsule, moving in continuity with the rectus femoris through flexion and extension. There was no obvious fatty atrophy or degeneration of the rectus femoris muscle belly.

**Figure 3:**
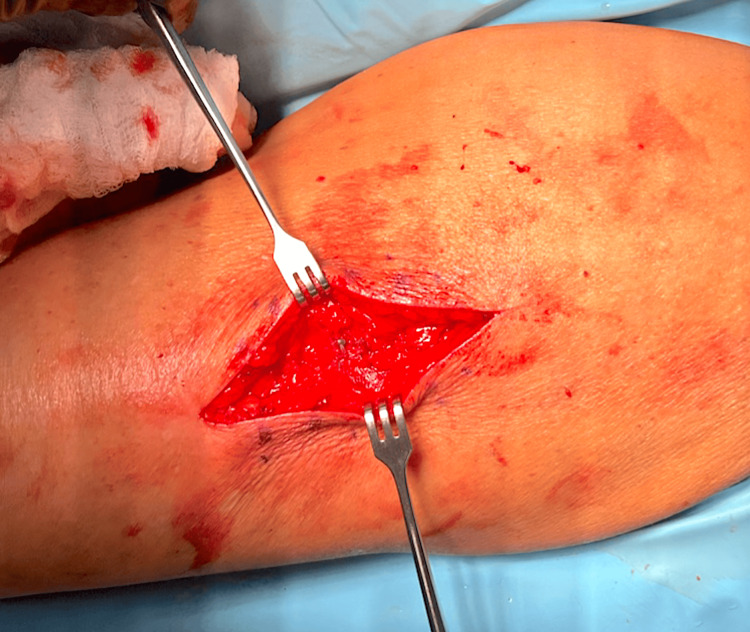
Closure of fascial defect without entrapment of underlying quadriceps

A 7 cm x 3 cm portion of the mass was sharply resected and sent to pathology for analysis. Grossly, it had the appearance of a lipoma. While depressing the quadriceps through the fascia to ensure it would not be entrapped by closure, a FiberWire® suture (Arthrex, Naples, Florida, United States) was used to close the fascial defect under appropriate tension (Figure 3). Pathology reported yellow glistening and homogenous soft tissue without areas of hemorrhage or necrosis, consistent with an intramuscular lipoma. The patient was seen at two, six, and 12 weeks follow-up with full resolution of pain and no further soft tissue bulge.

## Discussion

This report highlights a novel case in the literature of a large intramuscular lipoma of the anterior thigh with an associated overlying fascial defect. Clinically intramuscular lipomas typically present as a slow-growing, painless, asymptomatic mass. Deep and large lipomas may cause compression of adjacent peripheral nerves and soft tissue leading to pain, paresthesias, mechanical restrictions, and other compressive symptoms. Grossly, while intramuscular lipomas occur mostly in large muscles of the trunk and extremities, they can develop in any anatomical site [5-8]. In this case, a large intramuscular lipoma was found to be embedded in the rectus femoris with an overlying fascial defect identified, and resected due to pain with flexion and extension of the knee after failure of conservative measures.

It is important that large intramuscular lipomas be distinguished from lipomatosis and liposarcomas, which may also infiltrate the hypodermis, fascia, and muscle [3]. Lipomatosis affects younger patients, has multiple lipomas throughout the body, or involves the entire body region. It is often associated with various syndromes or obesity and presents with multiple poorly circumscribed lesions on MRI [3,9,10]. Furthermore, liposarcomas are often found to be heterogeneous and highly vascular, with irregularly thick septa [11].

To our knowledge, this is the only case of an infiltrative subfascial lipoma with involvement of the overlying fascia in terms of a fascial defect. Typically, fascial defects are caused by trauma or pre-existing constitutional weakness of the fascia, resulting in muscular hernias and activity restrictions. Diagnosis of fascial defects is typically made by dynamic ultrasound, but in cases of neoplasm such as in the current case, an MRI was warranted [12]. The exact etiology of this fascial defect is unknown, as there have been no prior reports of fascial defects associated with intramuscular lipomas; thus, we hypothesized the added pressure of the underlying lipoma predisposed this area to a fascial defect.

## Conclusions

This case highlights a novel presentation of a painful, infiltrative, intramuscular lipoma of the anterior thigh, with a large associated defect of the overlying fascia. MRI was helpful to exclude malignancy, and closure of the fascial defect without tension while maintaining free movement of the rectus femoris was important to ensure postoperative mobility.

## References

[1] Murphey MD, Carroll JF, Flemming DJ, Pope TL, Gannon FH, Kransdorf MJ (2004). From the archives of the AFIP: benign musculoskeletal lipomatous lesions. Radiographics.

[2] Fletcher CD, Martin-Bates E (1988). Intramuscular and intermuscular lipoma: neglected diagnoses. Histopathology.

[3] McTighe S, Chernev I (2014). Intramuscular lipoma: a review of the literature. Orthop Rev (Pavia).

[4] Chernev I (2014). Intramuscular lipoma: infiltrating vs. well-circumscribed variant. Pan Afr Med J.

[5] Bassett MD, Schuetze SM, Disteche C (2005). Deep-seated, well differentiated lipomatous tumors of the chest wall and extremities: the role of cytogenetics in classification and prognostication. Cancer.

[6] Pascua LR, León AA, Sánchez JA, Portal LF (2001). Intramuscular lipoma of the deltoid mimicking a sarcoma: a case report. Chir Organi Mov.

[7] Greenberg SD, Isensee C, Gonzalez-Angulo A, Wallace SA (1963). Infiltrating lipomas of the thigh. Am J Clin Pathol.

[8] Dutton JJ, Wright JD Jr (2006). Intramuscular lipoma of the superior oblique muscle. Orbit.

[9] Kamal D, Breton P, Bouletreau P (2010). Congenital infiltrating lipomatosis of the face: report of three cases and review of the literature. J Craniomaxillofac Surg.

[10] Lemaitre M, Aubert S, Chevalier B (2021). Rare forms of lipomatosis: dercum’s disease and roch-leri mesosomatous lipomatosis. J Clin Med.

[11] Kransdorf MJ, Bancroft LW, Peterson JJ, Murphey MD, Foster WC, Temple HT (2002). Imaging of fatty tumors: distinction of lipoma and well-differentiated liposarcoma. Radiology.

[12] Dyson K, Palan J, Mangwani J (2019). Bilateral non-traumatic lower leg fascial defects causing peroneal muscle herniation and novel use of a GraftJacket to repair the fascial defect. J Clin Orthop Trauma.

